# Mathematical formulation and parametric analysis of in vitro cell models in microfluidic devices: application to different stages of glioblastoma evolution

**DOI:** 10.1038/s41598-020-78215-3

**Published:** 2020-12-03

**Authors:** Jacobo Ayensa-Jiménez, Marina Pérez-Aliacar, Teodora Randelovic, Sara Oliván, Luis Fernández, José Antonio Sanz-Herrera, Ignacio Ochoa, Mohamed H. Doweidar, Manuel Doblaré

**Affiliations:** 1 Aragón Institute of Engineering Research (I3A), University of Zaragoza, Mariano Esquillor s/n, 50018 Zaragoza, Spain; 2 Institute for Health Research Aragón (IIS Aragón), Avda. San Juan Bosco, 13, 50009 Zaragoza, Spain; 3 Centro de Investigación Biomédica en Red en Bioingeniería, Biomateriales y Nanomedicina (CIBER-BBN), C/ Monforte de Lemos 3-5, Pabellón 11. Planta 0, 28029 Madrid, Spain; 4 School of Engineering, Department of Mechanics of Continuous Media and Theory of Structures, University of Seville, Camino de los descubrimientos, s/n, 41092 Sevilla, Spain

**Keywords:** Cancer models, Computational models, Applied mathematics

## Abstract

In silico models and computer simulation are invaluable tools to better understand complex biological processes such as cancer evolution. However, the complexity of the biological environment, with many cell mechanisms in response to changing physical and chemical external stimuli, makes the associated mathematical models highly non-linear and multiparametric. One of the main problems of these models is the determination of the parameters’ values, which are usually fitted for specific conditions, making the conclusions drawn difficult to generalise. We analyse here an important biological problem: the evolution of hypoxia-driven migratory structures in Glioblastoma Multiforme (GBM), the most aggressive and lethal primary brain tumour. We establish a mathematical model considering the interaction of the tumour cells with oxygen concentration in what is called the *go or grow* paradigm. We reproduce in this work three different experiments, showing the main GBM structures (pseudopalisade and necrotic core formation), only changing the initial and boundary conditions. We prove that it is possible to obtain versatile mathematical tools which, together with a sound parametric analysis, allow to explain complex biological phenomena. We show the utility of this hybrid “biomimetic in vitro-in silico” platform to help to elucidate the mechanisms involved in cancer processes, to better understand the role of the different phenomena, to test new scientific hypotheses and to design new data-driven experiments.

## Introduction

Biological processes integrate different cell populations, extracellular matrix, chemotactic gradients and physical cues, all conforming an extremely complex, dynamic and multiply interactive microenvironment^1–5^. Cells constantly adjust their function to accommodate the changing demands from the environment (e.g. oxygen and nutrients levels, substrate stiffness, drugs, etc.) with the objective of maintaining their intracellular and extracellular medium within a narrow range of physiological properties (homeostasis)^6^. As cells receive chemical and/or physical external stimuli, they modify their shape, location, internal structure and genomic expression, as well as their capacity to proliferate, migrate, differentiate, produce extracellular matrix or other biochemical substances, changing, in turn, the surrounding medium as well as sending new signals to other cells^7–9^. This two-way interaction between cells and environment is crucial in physiological processes such as embryogenesis, organ development, homeostasis, repair, and long-term evolution of tissues and organs among others, as well as in pathological processes such as atherosclerosis or cancer^10–13^. Understanding these mechanisms and interactions is therefore key to develop novel therapeutic strategies aiming at promoting (blocking) desirable (undesirable) events^14^.

As a consequence of this complexity, in vivo research (both in humans or animals) is very difficult due to the impossibility of controlling and isolate effects, or analysing specific situations due to ethical reasons. A simpler alternative is using in vitro experiments. A good reproduction of the particular biological process in vitro helps to better control the variables involved, and therefore to better understand the underlying mechanisms and interactions in specific physiological or pathological situations, as well as to provide tools for testing new drugs in a reliable way, reducing animal experiments. However, the predictive power of currently available in vitro models is still poor. This seems to be one of the main reasons for the continuous drop in the number of new drugs appearing yearly, dumping billion-dollar investments^15,16^. For example, despite structural three-dimensionality is one of the most important characteristics of biological processes^17^, cells are mostly cultured in the traditional Petri dish (2D culture), where cell behaviour is dramatically different from the observed in real tissues^18^. Recently, microfluidics has arisen as a powerful tool to recreate the complex microenvironment that governs tumour dynamics^19,20^. This technique allows reproducing important features that are lost in 2D cultures, as well as testing drugs in a much more reliable and efficient way^21–25^ .

Despite these more realistic and controlled conditions, it is still difficult, in in vitro experiments, to separate effects, check new scientific hypotheses, quantify the effect of each parameter or predict the outcome in *what if* situations. To overcome this limitation, a good possibility is combining the potential of new in vitro assays with the quantitative power and versatility of mathematical modelling and computational techniques^26,27^, particularly in the study of cancer^28–30^. Although mathematical modelling has demonstrated to be highly effective in many fields in Physics, Chemistry and Engineering, its ability to accurately represent reality in biological problems is still limited. The high dynamic complexity and non-linearity of the relations involved, the many highly-coupled interactions among different phenomena, the difficulty in identifying the initial state and the lack of data both for quantifying parameters and validating results, make the available models either too simple, or, on the contrary, too complex and cumbersome.

In many cases, models incorporate too many parameters, sometimes with unknown values or with a wide range of variation in literature (sometimes orders of magnitude) and with important hidden correlations^7,9,14^. The parameters are fitted to the particular data available, leading many times to trivial conclusions, mostly embedded in the model assumptions. This prevents the model to be useful for the whole family of similar problems, and the conclusions, results and parameters, difficult to generalise. Despite these strong limitations, in silico models, grounded in new biological knowledge, and driven by rigorous experimental and clinical data, have become invaluable tools to integrate knowledge across different biological scales, to perform quantitative analyses, and to test hypotheses in a cheap and fast way.

In cancer modelling, in particular, several results have been derived from mathematical approaches, quantifying, for example, the effect into tumour evolution of oxygen, biochemical molecules, ECM stiffness, or cell proliferation rate^31–33^ . In this work, we focus on glioblastoma multiforme (GBM), the most aggressive and lethal among the primary glioma tumours, and also the most frequent, accounting for 17% of all primary brain tumours^34^. Survival of patients with this type of tumour who undergo the first-line standard treatments (surgery followed by adjuvant chemotherapy and local radiation) has a median of 14 months since diagnosis and a 5-year survival rate of less than 10%^35^.

GBM progression is characterised by fast cell proliferation around blood vessels, eventually provoking their collapse, leading to hypoxia. Consequently, a necrotic core is formed around the vessel and the surviving cells migrate towards more oxygenated regions^36,37^, restarting the process of proliferation and creating waves of migrating tumour cells, which are known as pseudopalisades^36^ and appear in GBM histologies surrounding the necrotic core. This process of successive local hypoxia and cell migration has been proposed as one of the main driving forces of GBM invasion and aggressiveness^38^. There have been some attempts to build mathematical models to describe how these tumours grow and respond to therapies, both for in vitro experiments, and for in vivo models^39–44^. In Rejniak^44^, significant aspects, such as the importance of the hypoxic environment in the formation of cellular pseudopalisades^45^ and tumour vasculature (including angiogenesis and vessel cooption), the role of biophysical and biomechanical properties of the ECM in tumour cell invasion, or the role of microenvironmental niches and sanctuaries in the emergence of acquired drug resistance in tumours were reviewed. Other works focus on analysing the effect of mechanical cues in GBM evolution^10,12^.

In this paper, we address the problem of parameter analysis in the mathematical modelling of in vitro (microfluidic) cell processes associated with different stages of GBM evolution. We introduce a general framework in which the main cell processes involved (cell proliferation, differentiation, migration), all in response to the level of biochemical cues such as oxygen concentration, are mathematically formulated. This leads to a mechanistic model with a high number of parameters. Then, an extensive analysis of these parameters is made, both, from literature, and by correlating the associated in silico results with those derived from a specific microfluidic at-home lab assay: the appearance of auto-induced necrotic core far from the blood vessels in high-density cell regions^46^. We also analysed two additional configurations: local hypoxia inducing an oxygen gradient that forces GBM cells to migrate and proliferate with non symmetric (problem one) and symmetric (problem two) configurations. These processes are likely the responsible for cell fast migration from an occluded vessel and the subsequent pseudopalisade formation around another vessel, producing a new occlusion^36,45^. These experiments model therefore important scenarios of brain cancer evolution. The objective of this work is to demonstrate the potential of these mathematical models, if a proper parametric analysis is conducted, to get results close to the experiments with one single set of parameters obtained by fitting one single family of experiments, using the other two for validation purposes.

## Methods

### Experimental design

We refer to previous works in our group^45,46^ for a further explanation of the details of the experimental design. Briefly, human glioblastoma U251-MG cell line, purchased from Sigma Aldrich, was cultured in high glucose Dulbecco’s modified Eagle’s medium (DMEM) (Lonza, BE12-614F), supplemented with 10% foetal bovine serum (FBS) (Sigma, F7524), 2mM L-glutamine (Lonza, 17-605C) and penicillin/streptomycin (Lonza, 17-602E). U251-MG cells were stably transduced with green fluorescent protein (GFP)-expressing lentiviral vector, kindly provided by Dr. Prats, University Paul Sabatier, Toulouse, France^47^. Shortly, cells were incubated in 1:1 mixture of lentivirus suspension and Opti-MEM medium (Thermo, 31985062) supplemented with 5 μg/mL protamine sulfate (Sigma, P4505). After 24 h, the transduction medium was replaced with growth medium and the cells were routinely cultured for 2 weeks to remove the viral particles. Transduction efficiency was checked by fluorescence microscopy with more than 90% of the cells found to be EGFP-positive.

**Figure 1:**
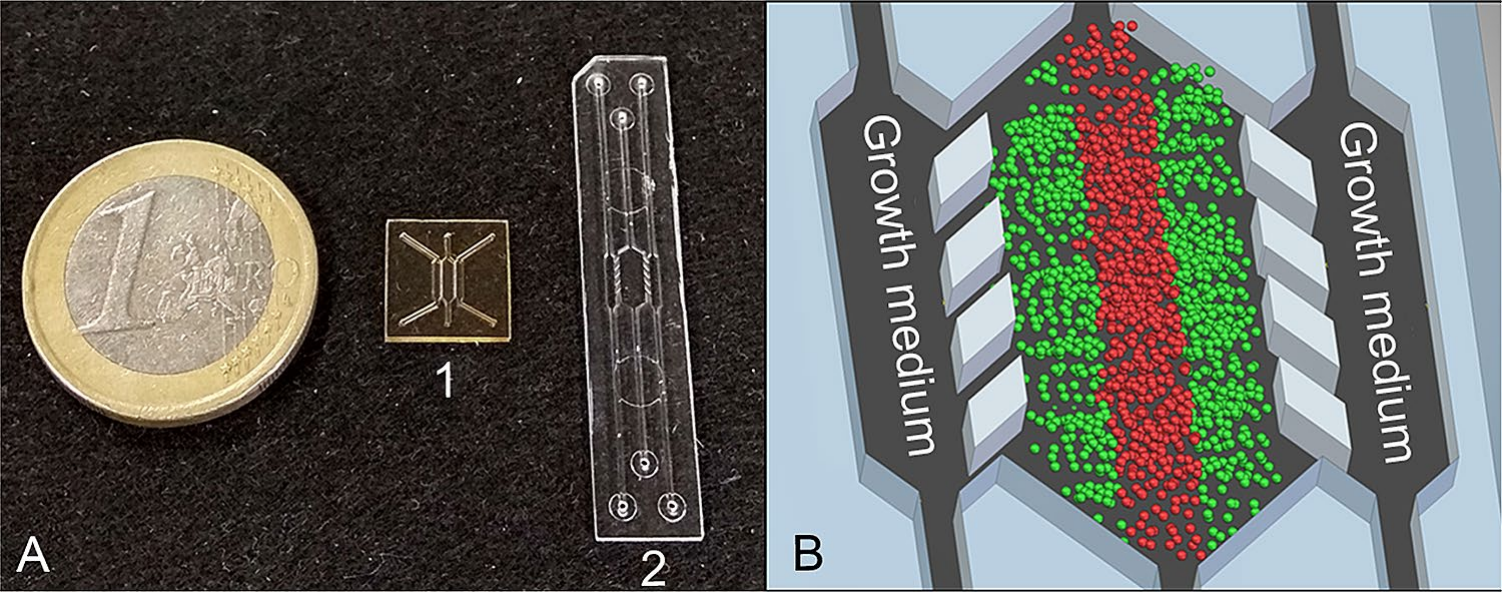
Description of the microdevice. (**A**) Microfluidic devices. A1—SU-8 device, A2—polystyrene/COP device. (**B**) Schematic view of the central region of the polystyrene/COP microdevice and necrotic core formation^46^.

In order to form a 3D structure, oxygen impermeable microfluidic devices (BEOnChip Ltd.) consisting of a central chamber and two lateral microchannels were used (Fig. 1). They had different dimensions and were made of SU-8, polystyrene or cyclic olefin polymer (COP), using different fabrication processes^45,46^. 3D distribution of cells was achieved within the central chamber, using collagen hydrogel. To prepare $100\; \mu \mathrm {L}$ of collagen hydrogel mixture with a $1.2\; \mathrm {mg/mL}$ final collagen concentration, $31.66\; \mu \mathrm {L}$ of $3.79\; \mathrm {mg/mL}$ collagen type I from rat tail (Corning, 354236), $0.79\; \mu \mathrm {L}$ of NaOH 1N (Sigma 655104), $10 \; \mu \mathrm {L}$ of DMEM 5x (Sigma D5523), $7.55 \; \mu \mathrm {L}$ of sterile distilled water and $50 \; \mu \mathrm {L}$ of cell suspension were mixed on ice. The mixture was well resuspended and injected into the central chamber of the microfluidic device using a micropipette. The hydrogel droplet was placed on the top of the inlet to prevent evaporation. The devices were placed into an incubator (37$^{\circ }$C, 5% $\mathrm {CO_2}$) for 15 min to promote collagen gel polymerization. Afterwards, pre-warmed growth medium was perfused through the lateral channels, mimicking blood vessels, and refreshed every 24h.

Laser confocal and fluorescence images were acquired at different focal planes within each microdevice using a Nikon Eclipse Ti-E C1 confocal microscope. Images were analysed using Fiji software (http://fiji.sc/Fiji). Fluorescence intensity was quantified, in accordance with the software instructions, by selecting a rectangular region across the central microchamber after creating the SUM projection image. In order to transform fluorescence intensity into cell concentration, the cell concentration is assumed proportional to the fluorescence intensity. The constant of proportionality is calculated assuming that the integral of the initial cell concentration along the chamber equals the total amount of cells.

**Figure 2:**
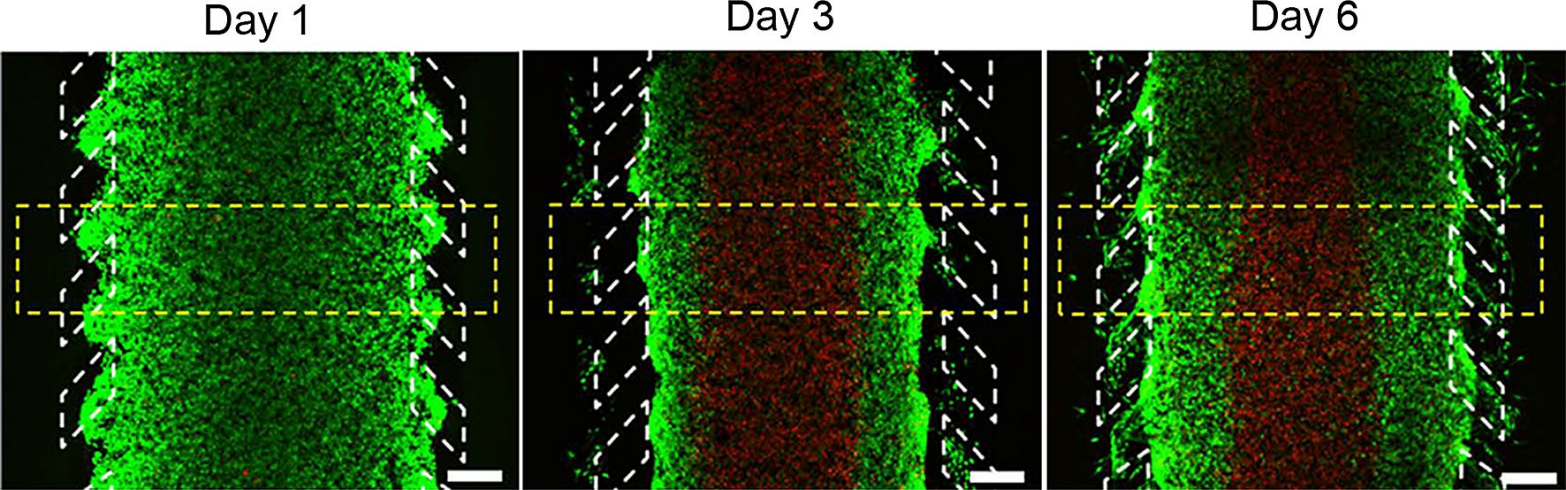
Necrotic core formation. U251 MG cells were seeded at a concentration of $40\times 10^6$ cells/mL within the central microchamber. Growth medium was perfused every day through the lateral channels. Viable cells were stained green with calcein AM and dead cells were labelled red with propidium iodide (modified from previous work^46^). Scale bar is $400 \; \mu \,\mathrm {m}$.

In order to produce the necrotic core formation, a high density of cells ($40\times 10^6 \mathrm {cells/mL}$) was embedded in the collagen hydrogel and injected within the central microchamber. Growth medium was refreshed every day and the culture was maintained for 6 days. Nutrients and oxygen are not able to reach the central part of the device due to cell consumption close to the microchannels, thus causing cell death in the central region appearing an autoinduced necrotic core (Fig. 2), mimicking the parts of the tumour far from functional blood vessels^46^. Visualisation of the necrotic core was performed by calcein/propidium iodide (CAM/PI) staining. Stock solutions of $1 \; \mathrm {mg/mL}$ CAM (Life Technologies, C1430) and $2 \; \mathrm {mg/mL}$ PI (Sigma P4170) were diluted to 2 and $4 \; \mu \mathrm {g/mL}$, respectively, in phosphate-buffered saline (PBS) (Lonza BE17-516F). CAM/PI solution was perfused through the lateral microchannels and incubated for 15 min. CAM becomes fluorescent once it reaches the cytoplasm of viable cells and PI stains dead cells, with destroyed membrane.

**Figure 3:**
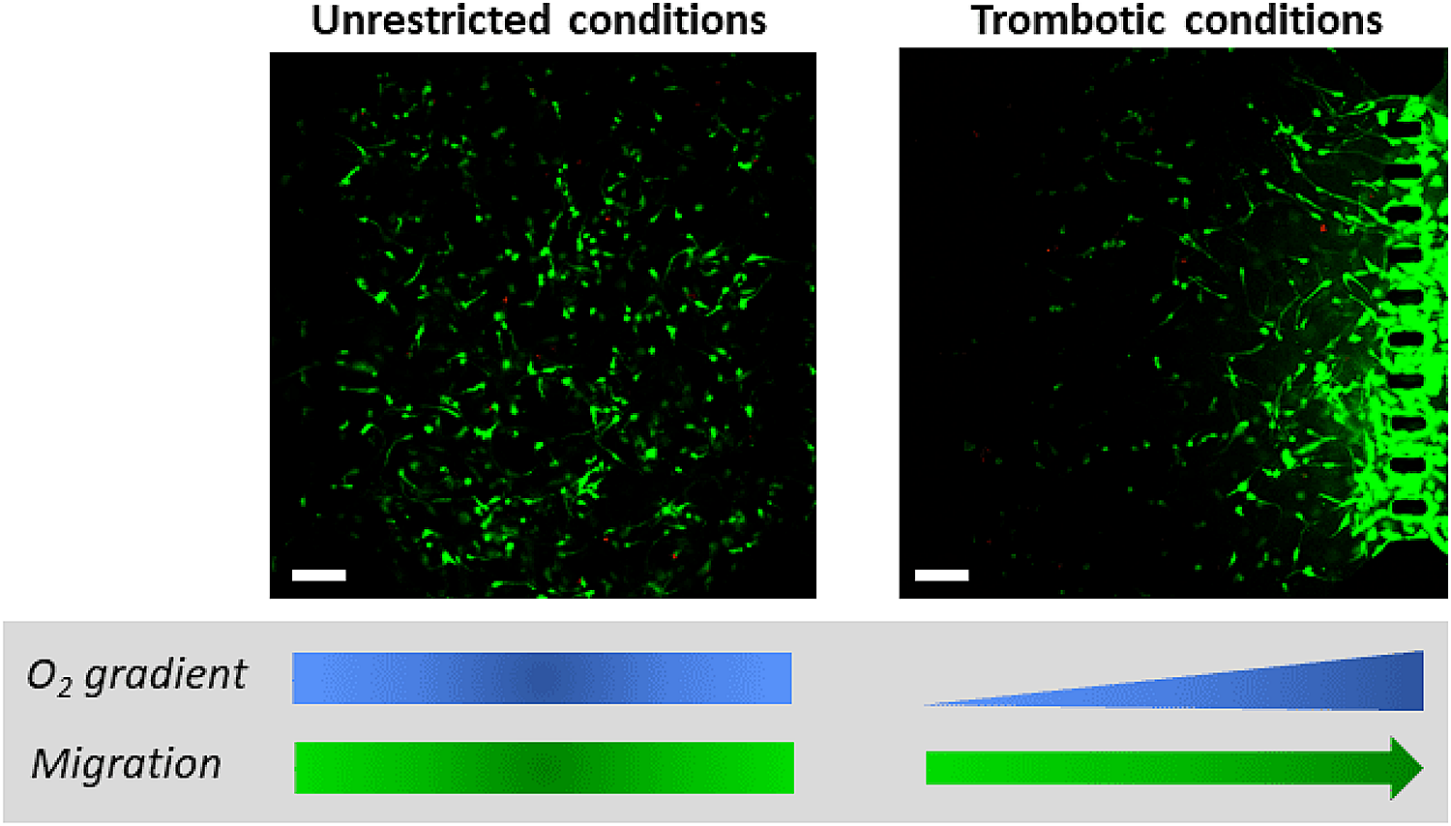
Pseudopalisade formation. U-251 MG cells at $4\times 10^6$ cells/mL were cultured within the microdevice. To mimic thrombotic conditions, medium flow was enabled to flow only through the right microchannel. Under unrestricted conditions, medium was refreshed once a day, through both lateral microchannels (modified from previous work^45^). Scale bar is $400 \; \mu \, \mathrm {m}$.

To promote pseudopalisade formation, cells were seeded at a low density ($4\times 10^6 \; \mathrm {cells/mL}$) within the central microchamber and one lateral channel was blocked, while constant medium flow was perfused through the other lateral channel. As the region next to the sealed channel was hypoxic, cells migrated towards the perfused channel, rich in nutrients and oxygen (Fig. 3). In the control device, both lateral channels were left open and migration was not observed^45^.

**Figure 4:**
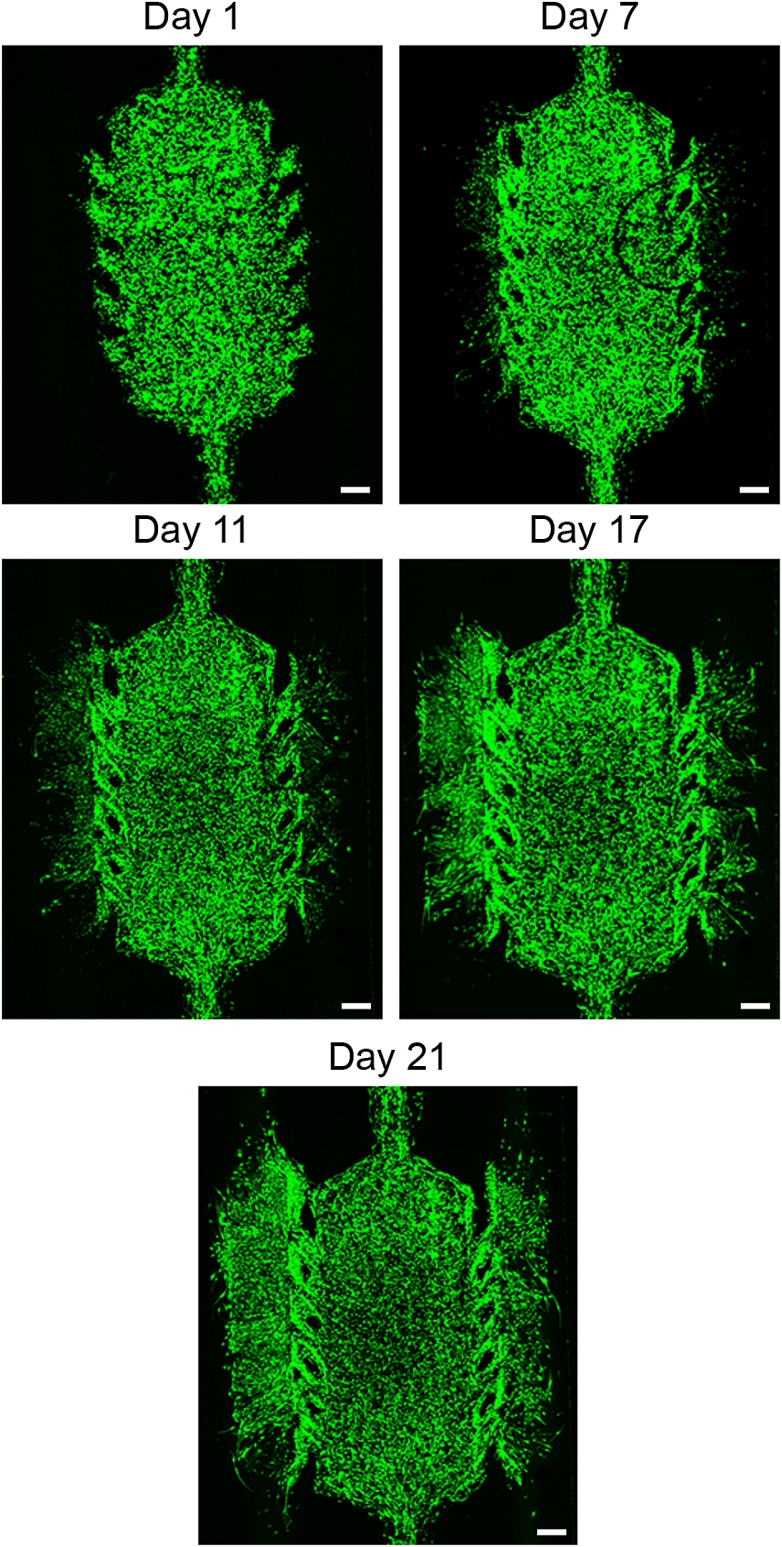
Double pseudopalisade formation. EGFP transduced U251-MG cells were embedded within the central microchamber at a concentration of $4 \times 10^6$ cells/mL. Growth medium was changed every day and the evolution of the cell culture over time was observed. Scale bar is $400 \; \mu \,\mathrm {m}$.

Finally, in the case of double pseudopalisade formation, cells were seeded again at low density ($4\times 10^6 \; \mathrm {cells/mL}$) within the central microchamber. In this case, the medium was perfused through both lateral channels and refreshed every day during 21 days. Hypoxic conditions in the centre of the microchamber induced cell migration towards the perfused channels and invasion of both of them (see Fig. 4).

### Mathematical methods

#### Model equations

Next, the mathematical model used here for modelling GBM evolution is presented. Even though the mathematical model can be defined for general multidimensional regime, due to the typology of the experiments and for simplicity, the problem may be approximated as one-dimensional, disregarding differences along the direction parallel to the lateral channels. We consider two cell phenotypes (dead cells and alive cells) interacting in the microfluidic device with one chemical species, i.e. oxygen, acting as a regulator of cell processes. These assumptions come from previous experiments in our group^46^ that showed that the distribution of other nutrients (glucose) is not responsible of changes in the cells configuration, being oxygen the main (and almost unique) stimulus for cell changes. The variable defining the number of cells for each population at each point and time is their respective concentration $u_i = C_i$ ($\frac{\mathrm {cells}}{\mathrm {mL}}$), $i=1,2$, where $i=1$ for alive cells and $i=2$ for dead cells. Similarly, we call $u_0 = O_2$ the continuum field of oxygen concentration (in $\mathrm {mmHg}$). Thus, we shall denote by ${\varvec{u}}=(u_0,u_1,u_2)^T$ the vector of field variables with 3 rows. The master equation that regulates each variable evolution is the transport equation including source terms:

1 $$\begin{aligned} \frac{\partial u_i}{\partial t} + \frac{\partial f_i}{\partial x}=s_i, \quad i=0,1,2. \end{aligned}$$

with $f_i$ the flow term that will include diffusion and chemotaxis for cells and diffusion for oxygen and $s_i$ the source term associated to production (proliferation) or loss (death of cells and consumption of oxygen). Note that Eq. (1) is, in general, nonlinear and should embed the coupling between the evolution of the different cell populations regulated by the oxygen concentration that may influence proliferation, migration and death, and oxygen consumption kinetics.

In our case, the cell flow term depends on the “random” movement of cells, only driven by differences in their concentration, that is, a diffusion term, and the chemotaxis induced by differences in oxygen concentration (oxygen gradient). For the oxygen pressure, only the diffusion was considered. Then,

2 $$\begin{aligned} f_i = -D_i\frac{\partial u_i}{\partial x}+K_i u_i\frac{\partial u_0}{\partial x}, \quad i=0,1,2. \end{aligned}$$

where $D_i = D_i({\varvec{u}})$ is the diffusion coefficient and $K_i = K_i({\varvec{u}})$ the chemotaxis coefficient for population *i*. Recall that $K_0 = 0$. In Eq. (2), we have considered that both coefficients may depend, in general, on the local densities of each cell phenotype and the local concentration of oxygen.

Taking into account the two effects already mentioned for the source term of proliferation and differentiation in cell concentrations and consumption in oxygen, we can write:

3 $$\begin{aligned} s_0= & {} -\sum _{j=1}^2 \alpha _j u_j \end{aligned}$$

4 $$\begin{aligned} s_i= & {} \frac{1}{\tau _{ii}}u_i-\sum _{j=1,j \ne i}^2\frac{1}{\tau _{ij}}u_i+\sum _{j=1,j \ne i}^2\frac{1}{\tau _{ji}}u_j, \quad i=1,2. \end{aligned}$$

where $\alpha _{j}=\alpha _{j}({\varvec{u}})$ is the oxygen rate consumed by the cell population *j*, $\tau _{ii}=\tau _{ii}({\varvec{u}})$ is the characteristic time of proliferation for population *i* and $\tau _{ij}=\tau _{ij}({\varvec{u}})$ the characteristic time of differentiation from population *i* to population *j*, that, again, and in general, may depend on the chemical conditions, as well as the local densities of cells. Recall that the apoptotic or necrotic processes are included here as specific differentiation types to the specific phenotype of dead cells.

Equation (1) has to be complemented with the corresponding boundary conditions. We assume here the general case of Robin-like boundary conditions, that is:

5 $$\begin{aligned} I_i(x^*,t)\left( u_i-g_i(x^*,t)\right) +J_i(x^*,t)\left( f_i-h_i(x^*,t)\right) = 0, \quad i=0,1,2. \end{aligned}$$

In the previous equation, $x^*=0,L$, where *L* is the width of the chamber. $I_i= I_i(x^*,t)$ and $J_i = J_i(x^*,t)$ are functions characterising the boundary permeability to cell movement or oxygen flow through the boundary, and $g_i(x^*,t)$ and $h_i(x^*,t)$ functions defining the controlled value of cell or oxygen concentration and flux at the boundaries. Note that, if $I_i = 1$ and $J_i=0$, we have Dirichlet boundary conditions (cell population concentration prescribed at the boundary) and, if $I_i = 0$ and $J_i = 1$, we have Neumann boundary conditions.

Finally, the initial conditions for oxygen and each cell population concentration have to be defined:

6 $$\begin{aligned} u_i(x,t=0) = u^0_i(x), \quad i=0,1,2. \end{aligned}$$

where $u_i^0(x)$ is a known function.

In order to particularise the general equations presented for modelling the population and species evolution in the in vitro experiments made on GBM cells, it is necessary to choose a functional relationship between the coefficients of the model, that is, $D_i$, $i=0,1,2$, $K_i$, $\tau _{ij}$, $i,j=1,2$ and $\alpha _j$, $j=1,2$, and the field variables ${\varvec{u}}$.

Even though some papers consider three^48^ or four^45^ cell phenotypes, here, only two phenotype populations (alive and dead cells) have been considered, thus disregarding possible changes in phenotype along the duration of our experiments. This does not mean that all cells in the chamber equally proceed in terms of proliferation, migration or oxygen consumption, since all these processes depend as well on the particular conditions of the surrounding environment, but that all cells respond equally when they are subjected to the same local environmental conditions. We consider with this assumption that cell adaptation requires longer periods under stressing conditions to modify permanently their internal machinery. Another reason for this assumption is that, in absence of gene expression techniques, it is impossible to distinguish between differentiation into a different phenotype or a change in the cell behaviour as reaction to environmental changes, so, considering one single phenotype for alive cells results in fewer parameters and a better understanding of the role of the different phenomena and parameters, an easier calibration and less uncertainty.

In our microfluidic device, with a controlled production of the hydrogel, we can assume that it is homogeneous and remains with the same properties all along the experimental or, alternatively, that the potential changes in those properties do not affect significatively the cell properties nor the oxygen diffusivity. For alive cells, migration is split in oxygen mediated chemotaxis and pedesis. Dead cells are considered as an inert population ($D_2 = K_2 = \frac{1}{\tau _{21}} = \frac{1}{\tau _{22}} = \alpha _2 = 0$). Besides, growth and death rates are also assumed to be dependent on nutrients and oxygen environment. With all these assumptions, it is possible to consider a functional dependency for the following parameters:

7 $$\begin{aligned} D_0&= D_{O_2} \nonumber \\ D_1&= D_n \nonumber \\ K_1&= \chi F_{\mathrm {chemo}}(C_1)\Pi _{\mathrm {chemo}}(O_2) \nonumber \\ \frac{1}{\tau _{11}}&= \frac{1}{\tau _{g}}F_{\mathrm {gr}}(C_1,C_2)\Pi _{\mathrm {gr}}(O_2) \nonumber \\ \frac{1}{\tau _{12}}&= \frac{1}{\tau _{d}}\Pi _{\mathrm {ap}}(O_2) \nonumber \\ \alpha _1&= \alpha F_{\mathrm {kin}}(O_2) \nonumber \\ \end{aligned}$$

Functions *F* are nonlinear corrections for cell growth, cell death and oxygen consumption kinetics, while $\Pi$ functions model how the different cell mechanisms are activated depending on the oxygen level, $D_{O_2}$ is the oxygen diffusion coefficient, $D_{n}$ is the diffusion of the normoxic cell population coefficient, $\chi$ is the normoxic cell population chemotaxis coefficient, $\tau _g$ is the characteristic proliferation time, $\tau _d$ is the death characteristic time and $\alpha$ is the oxygen consumed per unit time and cell. Since cell populations adapt their behaviour to oxygen supply and space availability, two major corrections should be considered in the migration term:

- Cellular motility is only possible when the surrounding tissue is not cell saturated^49^.
- Migration following the oxygen gradient happens only when the oxygen supply is below a critical threshold, activating the cell motility mechanism^50,51^.

According to these two major assumptions, a rectified linear unit (ReLU) kind activation function^52^ was here used to take into account each of the two phenomena, so the chemotaxis corrections may be written as:

8 $$\begin{aligned} F_{\mathrm {chemo}}(C_1)&= \phi _{-}(C_1;C_{\mathrm {sat}}) \nonumber \\ \Pi _{\mathrm {chemo}}(O_2)&= \phi _{-}(O_2;O_2^H) \end{aligned}$$

with

9 $$\begin{aligned} \phi _{-}(x;\theta )= \left\{ \begin{array}{lcc} 1 &{} \mathrm {if} &{} x \le 0 \\ 1-\frac{x}{\theta } &{} \mathrm {if} &{} 0 \le x \le \theta \\ 0 &{} \mathrm {if} &{} x > \theta \end{array} \right. \end{aligned}$$

where $\theta$ is a threshold parameter.

Here $O^{H}_2$ is the hypoxia-induced migration activation threshold, representing the oxygen level below which cell migration is activated and $C_{\mathrm {sat}}$ is the cell saturation concentration.

The proposed model is in line with the *go or grow* dichotomy established in GBM literature^53^. Cell energetic resources are spent either in cell migration or in cell proliferation. However, cell proliferation also depends on other needs as nutrient supply or availability of space to grow and split. According to this, we propose a model combining logistic growth and the *go or grow* paradigm based on oxygen supply. We define the growth corrections as:

10 $$\begin{aligned} \Pi _{\mathrm {gr}}(O_2)&= \phi _{+}(O_2;O_2^H) \nonumber \\ F_{\mathrm {gr}}(C_1,C_2)&= \rho (C_1+C_2;C_{\mathrm {sat}}) \end{aligned}$$

with

11 $$\begin{aligned} \phi _{+}(x;\theta )= \left\{ \begin{array}{lll} 0 &{} \mathrm {if} &{} x \le 0 \\ \frac{x}{\theta } &{} \mathrm {if} &{} 0 \le x \le \theta \\ 1 &{} \mathrm {if} &{} x > \theta \end{array} \right. \end{aligned}$$

and $\rho$ is the logistic correction factor:

12 $$\begin{aligned} \rho (x;\theta ) = 1-\frac{x}{\theta } \end{aligned}$$

The function $\Pi _{\mathrm {gr}}$ is responsible of the *go or grow* dichotomy and the second is the logistic model for cell population growth.

Cell death is a natural process depending on many factors and agents and has an inherent stochastic nature^54^. Anoxia is one fundamental cause of cell death^55^. Here, a two-parameter sigmoid model is used, able to capture necrosis and apoptosis phenomena:

13 $$\begin{aligned} \sigma _{-}(x;\theta ,\Delta \theta )=\frac{1}{2}\left( 1-\mathrm {tanh}\left( \frac{x-\theta }{\Delta \theta }\right) \right) \end{aligned}$$

where $\theta$ is a threshold parameter and $\Delta \theta$ is a sensitivity parameter. They can be seen as a pair of location-spread parameters summarising the stochastic behaviour of the considered phenomenon. With this notation:

14 $$\begin{aligned} \Pi _{\mathrm {ap}}(O_2) = \sigma _{-}(O_2;O^{A}_2,\Delta O^{A}_2) \end{aligned}$$

with $O^{A}_2$ and $\Delta O^{A}_2$ the location and spread parameters associated with the oxygen concentration inducing cell death.

Finally, oxygen consumption is a complex phenomenon related to the oxidative phosphorylation that occurs in the membrane of cellular mitochondria^56^. Many authors have considered a zero-order consumption function, i.e. a constant consumption rate independent of oxygen concentration $O_2$^57–59^. A more realistic assumption is to describe the consumption function using the Michaelis–Menten model for enzyme kinetics^48,60^. This model is a correction of the linear consumption and states that:

15 $$\begin{aligned} r(x;K) = \frac{x}{x+K} \end{aligned}$$

where *K* is a model parameter. This type of equation was observed for the oxygen consumption rate in the late 1920s and early 1930s^61^. This equation describes more accurately the consumption at low oxygen concentrations and is compatible with previous constant consumption rate models, thus allowing the possibility of comparison with previous studies.

Using this notation and the one introduced in our mathematical formulation, we can write:

16 $$\begin{aligned} F_{\mathrm {kin}}(O_2) = r(O_2;O_2^{(T)}) \end{aligned}$$

where $O_2^{(T)}$ is the oxygen concentration at which the reaction rate is half of the rate in a fully oxygenated medium, therefore related to the oxidative phosphorylation kinetics, and the cell structure and morphology (size and number of mitochondria, etc.) and the diffusion process in the cytoplasm.

In the microfluidic device, the culture chamber is connected to the oxygen supplying channels by means of small cavities. The volume and the number of these cavities depend on the microfluidic device design and they are directly related with potential cell losses during the experiment. Actually, when cell populations arrive to the interface between these cavities, some of them may reach the channel and leave the culture. To take into account this phenomenon, we have considered Robin boundary conditions. In principle, since both sides have the same design (number and width of the interface microcavities) there is no reason for considering differences in cell losses (in percentage) between both sides. Therefore, as there is no cell supply through the lateral channels the boundary condition writes:

17 $$\begin{aligned} u_1(x^*,t) + J_1f_1(x^*,t) =0 \end{aligned}$$

With regard to the dead cell population, homogeneous Neumann boundary conditions are considered since this population does not migrate neither by diffusion nor chemotaxis, so we have

18 $$\begin{aligned} u_2(x^*,t) =0 \end{aligned}$$

Regarding the oxygen supply, we shall consider two possibilities, associated to two different conditions: when oxygen is supplied normally, Dirichlet boundary conditions are considered, that is, we shall assume that the oxygen concentration at the channels remains constant and known throughout the experiment, that is:

19 $$\begin{aligned} u_0(x^*,t) =O_2^{*} \end{aligned}$$

where $O_2^{*}$ is a known value.

On the other hand, when a channel is sealed, we assume that oxygen provision is negligible, so Neumann boundary conditions are considered:

20 $$\begin{aligned} f_0(x^*,t) = 0 \end{aligned}$$

Finally, we assume that, at time $t=0$, all cells are alive and the cell population concentration is known throughout the whole culture chamber. That is, $C_1(x,t=0) = C_1^0(x)$ is known (measured experimentally) and $C_2(x,t=0) = 0$. Moreover, the oxygen profile is assumed to be constant along the chamber and equal to the concentration in the channels, due to the small characteristic time of oxygen diffusion within the hydrogel compared to the characteristic time of cell processes:

21 $$\begin{aligned} u_0(x,t=0) = O_2^* \end{aligned}$$

The differential equation (1) with boundary (5) and initial (6) conditions results in a nonlinear parabolic differential equation in time, with only one space dimension. This equation was solved here numerically by means of a time-space integrator based on a piecewise nonlinear Galerkin approach which is second-order accurate in space^62^, and compatible with this kind of nonlinear equations and boundary conditions. The domain length (associated with the microfluidic device) and mesh size used for the simulation of each experiment are summarised in Table 1.

**Table 1:** Domain and mesh size for the different simulations.

Experiment	Chamber length $L \, [\mu m]$	Mesh size $\Delta x \, [\mu m]$
Necrotic core formation	2000	3.0
Pseudopalisade formation	916	4.8
Double pseudopalisade formation	2897	12.0

## Results

### Parametric analysis

In this section, we discuss the values used in literature for each of the parameters in our model (described in Methods section). We found that many of them are essentially unknown or with high ranges of variation. Our effort goes in the direction of discriminating which works define some of these parameters in similar conditions and trying to identify the most likely values within the intervals identified in the literature.

#### Bibliographic review

In the available literature it is difficult to identify the precise values of such parameters, due to the diversity of models and experimental conditions. Consequently, we include this review clarifying the process for the parameters definition or calculation, often after a reference-crossing process.

**Cell diffusion coefficient** ($D_n$). The cell diffusion coefficient is a parameter related to the undriven cellular motility. Cell motility is frequently evaluated in experimental works from a global point of view, that is, including random motility and hypoxia-induced chemotaxis. In this work, however, both phenomena are taken into account separately so diffusion acts as a pure regularisation term while chemotaxis is the main driving force in cell migration. Therefore, only diffusion coefficients associated with healthy tissues in perfect oxygenation conditions will be taken into account.

According to Tija et al.^63^, this parameter depends on the substrate mechanical properties. For a standard collagen ECM, similar to the culture hydrogel here used, a value of $1 \times 10^{-9} \, \mathrm {cm^2}/s$ is proposed. Martínez-González et al. propose in one of their works^48^ a value of $6.6 \times 10^{-12} \, \mathrm {cm^2/s}$, while in another^64^ a value of $5 \times 10^{-10} \, \mathrm {cm^2/s}$ is assigned, one order of magnitude lower than the mean of the values reported by Rockne et al.^65^ ($5\times 10^{-9} \, \mathrm {cm^2/s}$). Wang et al.^66^ discuss this value for different locations in the brain, observing that glioma cells migrate quicker in white matter than in grey matter, highlighting also the important variation of this coefficient with the tumour stage and after chemotherapy and radiotherapy, ranging all values from $3 \times 10^{-7} \, \mathrm {cm^2/s}$ to $5 \times 10^{-5} \, \mathrm {cm^2/s}$ (median of $3 \times 10^{-6} \, \mathrm {cm^2/s}$). Hathout et al.^67^, use values from $5 \times 10^{-7} \, \mathrm {cm^2/s}$ to $2 \times 10^{-6} \, \mathrm {cm^2/s}$ during the tumour initial state.

**Chemotaxis coefficient** ($\chi$). This coefficient is difficult to estimate when considering chemotaxis as an isolated phenomenon^68^. Ford et al.^69^ define $\chi = \chi _0f(O_2)$ with $\chi _0$ ranging from $1.5 \times 10^{-5} \, \mathrm {cm^2/s}$ to $7.5 \times 10^{-4} \, \mathrm {cm^2/s}$ depending on the complex affinity while several expressions are proposed for *f*. For example, a hyperbolic tangent dependence is presented, based on a probabilistic mechanobiological model for individual bacteria^70^.

Many other works define the chemotaxis coefficient with respect to the normalised concentration $\frac{O_2}{O_2^v}$ where $O_2^v$ is the vessel oxygen supply pressure. Agosti et al.^68^ assume a value of $1.5 \times 10^{-4} \, \mathrm {cm^2/(mM\cdot s)}$ for an oxygen concentration in vessels $O_2^v$ of $0.07 \, \mathrm {mM}$^71^. Therefore, $\chi$ is of the order $2 \times 10^{-7} \, \mathrm {cm^2/(mmHg \cdot s)}$ assuming an oxygen supply in vessels of $40-60 \, \mathrm {mmHg}$^72, 73^. With the same conversion between oxygen concentration and pressure, a value between $3 \times 10^{-10} \mathrm {cm^2/(mmHg \cdot s)}$ and $1 \times 10^{-9} \mathrm {cm^2/(mmHg \cdot s)}$ is adopted by Agosti et al. in their work^74^. Finally, Bearer et al.^75^ propose a chemotaxis coefficient of $10^5 \, \mathrm {\mu m^2/d}$, which gives an equivalent value of $2 \times 10^{-10} \mathrm {cm^2/(mmHg \cdot s)}$.

**Hypoxia-induced migration activation threshold** ($O_2^H$). In our model, hypoxia induced cell migration is relevant only when the oxygen pressure is under a certain threshold $O_2^{th}$. According to previous works on GBM simulation^48,64^, cells are considered under hypoxia conditions, when the oxygen pressure is under $7 \, \mathrm {mmHg}$ (approximately $12 - 18 \%$ of the blood vessel oxygen pressure). Agosti et al. consider in one work^68^ a threshold of $15 - 50\%$ of blood vessel oxygen pressure and later^74^ a threshold of $30\%$ is used. In the review paper^76^, a ratio of $12-25\%$ between healthy and tumorous tissue oxygen pressure is considered.

**Growth characteristic time** ($\tau _g$). This is also a very context-dependent parameter, since the cell metabolism highly varies between cell types and individuals. In addition, our proposed logistic model implies that the measured growth time in the particular experimental conditions depends on the cell concentration, and therefore could vary with considered values reported in literature. Nevertheless, some growth characteristic times reported in literature for logistic, exponential or Gompertz growth models are here discussed. Gerlee et al.^77^ consider a growth time of $16 \, \mathrm {h}$ for a cell automaton model, based on a previous work^78^. Other authors propose a value of $24 \, \mathrm {h}$^79,80^ using an exponential model (so the growth characteristic time is underestimated). A logistic model is used by Agosti et al.^68^, with a characteristic time between $48 \, \mathrm {h}$ and $2000 \, \mathrm {h}$, closer to the values obtained by Wang et al.^66^ (with median $408 \, \mathrm {h}$) and by Rockne et al.^65^ (mean of $450 \, \mathrm {h}$, using MRI techniques). In other works by Agosti et al. a value of $300 \, \mathrm {h}$ is proposed^74^ based on a Gompertz growth model^81^. Among the 36 tumours simulated by Hathout et al.^67^ a range between $240 \, \mathrm {h}$ and $1200 \, \mathrm {h}$ was used. Finally, Martínez–González et al.^64^ propose values between $336 \, \mathrm {h}$ and $576 \, \mathrm {h}$ using a Fisher–Kolmogorov approximation, and later^48^ values between $24 \, \mathrm {h}$ and $48 \, \mathrm {h}$ based on experimental studies^82–84^ are considered.

In our work, based on the *go or growth* assumption, the growth characteristic time is infinite in absence of oxygen and decreases until the oxygen concentration exceeds the hypoxia threshold. Thus, our model captures this variability from hypoxic to normoxic media, where growth is accelerated and therefore characteristic times are smaller.

**Cell concentration saturation** ($C_{\mathrm {sat}}$). An important variability is found in the literature when referring to this parameter, with a range that covers several orders of magnitude. For example, Rockne et al.^65^, propose a value of $10^{11}\, \mathrm {cell/cm^3}$ whereas Hathout et al.^67^ use the value of $10^8\, \mathrm {cell/cm^3}$ according to previous experimental works^85^.

This parameter depends on the mechanical and structural properties of the medium and on nutrients supply so its variability is natural. In any case, it does not have a major impact in simulations for cell concentrations much lower than the saturation capacity.

**Death characteristic time** ($\tau _d$). Even in the case where no cell-concentration dependence is considered, death characteristic time also varies between studies since it is directly measured, without considering, for example, oxygenation conditions, as for the growth characteristic time. In the automaton model from Gerlee et al.^77^, an average apoptosis probability of 0.18 is obtained, resulting in a death characteristic time of $72 \, \mathrm {h}$ as proposed by Frieboes et al.^80^. In their works, Agosti et al. propose values between $160 \, \mathrm {h}$ and $400 \, \mathrm {h}$^68^, and of $600 \, \mathrm {h}$^74^. Finally, Martínez–González et al.^48,64^ use two different phenotypes to model the tumour population (normoxic and hypoxic), but they assume that once the cell has arrived to hypoxic conditions, its death characteristic time is $48 \, \mathrm {h}$^48^ or $7 \, \mathrm {d}$^64^.

We model death with a sigmoid function, which integrates both death causes: apoptosis, that is mainly stochastically mediated and necrosis, induced by oxygen lack. This model explains better the variability found in literature, ranging from $72 \, \mathrm {h}$ in anoxia to $600 \, \mathrm {h}$ in normoxia, via the two parameters regulating cell switch, discussed below.

**Anoxia-induced death location parameter** ($O_2^A$). Even though in many mathematical models it is assumed that the hypoxia threshold, inducing migration or proliferation (and therefore the fundamental parameter explaining the *go or grow* dichotomy), and the anoxia threshold (as an indicator of necrosis) are the same^68,74^; whereas other authors distinguish between both phenomena. Martínez-González et al.^48,64^ select a value of $0.7 \, \mathrm {mmHg}$ for the anoxia level, as explained in previous works^86^, corresponding to approximately $1-2\%$ of vessel oxygen concentration ($40-60 \, \mathrm {mmHg}$). Vital-López et al.^71^, consider that with $15\%$ of normal concentration ($12 \, \mathrm {mmHg}$ in brain^76^), the death probability has a value of $50\%$, resulting in a value of $1.8 \, \mathrm {mmHg}$.

**Anoxia-induced death spread parameter** ($\Delta O_2^A$). This parameter illustrates the variability of the cell death phenomenon. High values of $\Delta O_2$ are related to very random death, that is, apoptosis mediated by other effects not considered in this model, whereas low values of $\Delta O_2$ are related to death dominated by necrosis, i.e. death only occurs when cells are under the anoxia threshold. Martínez-González et al.^48,64^ adopt a value of $0.1 \, \mathrm {mmHg}$ while Vital-López et al.^71^ consider a dimensionless slenderness parameter of $s=200$ which turns into $3 \, \mathrm {mmHg}$ when considering our model, thus considering a higher variability in cell death rate.

**Oxygen diffusion coefficient** ($D_{O_2}$). The oxygen diffusion coefficient is classically known to be around $10^{-5}\, \mathrm {cm^2/s}$ at $37^\circ \mathrm {C}$. Daşu et al.^87^ propose a value of $2 \times 10^{-5}\, \mathrm {cm^2/s}$ according to previous studies^57,58^ that assign an intermediate value between oxygen diffusion in water and muscle^88^. Other works^77,89^ use a value of $1.8 \times 10^{-5}\, \mathrm {cm^2/s}$. Recent computational patient-specific studies^74^ assume a value of $10^{-5}\, \mathrm {cm^2/s}$. It is important to note that, in the present work, hydrogels used in microfluidic devices try to reproduce soft human tissue, so similar values can be used.

**Oxygen consumption rate** ($\alpha$). The maximum value of $\alpha$ is much debated^76,90–92^ ranging from $2 \, \mathrm {\mu L/(min \cdot g)}$^76^ to $55 \, \mathrm {\mu L/(min \cdot g)}$^90^. There are several possible explanations for this large range of values reported as explained by Daşu et al.^87^, such as the influence of the tissue metabolic characteristics in the consumption rate, the variations of the temperature and pressure conditions when measuring the oxygen volume or experimental reasons associated with the measuring method. The most often quoted value is $15 \, \mathrm {mmHg/s}$ for the maximum consumption rate in healthy tissue^57,87,93^. This consumption rate gives a maximum diffusion distance of $143 \, \mathrm {\mu m}$^93^, for a blood vessel with $40 \, \mathrm {mmHg}$. Assuming that a healthy tissue has a concentration of $0.2C_{\mathrm {sat}}$^64^, we obtain $7.5 \times 10^{-7} \, \mathrm {(mmHg \, cm^3)/(cell \, s)}$. Assuming the same ambient cell concentration, using the value proposed by Agosti et al.^68,74^ we obtain $2.5 \times 10^{-7} \, \mathrm {(mmHg \, cm^3)/(cell \, s)}$. The consumption selected for the automaton presented by Gerlee et al.^77^, based on studies on GBM spheroids^94^, is fixed to $2.3 \times 10^{-16} \, \mathrm {mol/(cell \, s)}$, equivalent to $1.4 \times 10^{-8} \, \mathrm {(mmHg \, cm^2)/(cell \, s)}$ assuming an oxygen background concentration of $c_0 = 1.7 \times 10^{-8} \, \mathrm {mol/cm^2}$. A value of $4 \times 10^{-17} \, \mathrm {mol/(cell \, s)}$ is obtained from the data analysed by Griguer et al.^95^ resulting in $2.5 \times 10^{-9} \, \mathrm {(mmHg \, cm^2)/(cell \, s)}$.

**Michaelis–Menten constant** ($O_2^T$). According to Daşu et al.^87^, the $O_2^{(T)}$ constant seems to have little influence on the diffusion at high $O_2$ concentrations and therefore we use a value of $O_2^{(T)} = 2.5 \, \mathrm {mmHg}$, equal to the hypoxic threshold often used in the Alper and Howard-Flanders equation describing the oxygen enhancement ratio^96^. This value has been chosen in recent simulation models^48,64^.

In Table 2 all numerical parameters of the mathematical model are shown with their corresponding variation range extracted from the bibliography.

**Table 2:** Range of variability of the parameters in the bibliography.

	Minimal value	Maximal value	Units
$D_n$	$6.6 \times 10^{-12}$ (Martínez-González et al.^48^)	$5.0 \times 10^{-5}$ (Wang et al.^66^)	$\mathrm {cm^2/s}$
$C_{\mathrm {sat}}$	$1.0 \times 10^8$ (Hatout et al.^67^)	$1.0 \times 10^{11}$ (Rockne et al.^65^)	$\mathrm {cell/mL}$
$\chi$	$2.0 \times 10^{-10}$ (Bearer et al.^75^)	$7.5 \times 10^{-4}$ (Ford et al.^69^)	$\mathrm {cm^2/mmHg \cdot s}$
$\tau _g$	$5.8 \times 10^4$ (Gerlee et al.^77^)	$7.2 \times 10^6$ (Agosti et al.^68^)	$\mathrm {s}$
$\tau _d$	$1.7 \times 10^5$ (Martínez-González et al.^48^)	$2.2 \times 10^6$ (Agosti et al.^74^)	$\mathrm {s}$
$D_{O_2}$	$1.0 \times 10^{-5}$ (Agosti et al.^74^)	$2.0 \times 10^{-5}$ (Daşu et al.^87^)	$\mathrm {cm^2/s}$
$\alpha$	$2.5 \times 10^{-9}$ (Griguer et al.^95^)	$7.5 \times 10^{-7}$ (Martínez-González et al.^64^)	$\mathrm {mmHg \cdot mL/cell\cdot s}$
$O_2^T$	2.5 (Daşu et al.^87^)	2.5 (Daşu et al.^87^)	$\mathrm {mmHg}$
$O_2^H$	7.0 (Vaupel et al.^76^)	30 (Agosti et al.^68^)	$\mathrm {mmHg}$
$O_2^A$	0.7 (Brown et al.^86^)	1.8 (Vital-López et al.^71^)	$\mathrm {mmHg}$
$\Delta O_2^A$	0.1 (Martínez-González et al.^48^)	3.0 (Vital-López et al.^71^)	$\mathrm {mmHg}$

#### Model parameter fitting

The value of each parameter was initially chosen to stay within the ranges found in the bibliography (Table 2). In order to calibrate the specific value for each parameter, the formation of the necrotic core experiment (described in Methods section) was selected as case study. Robin boundary conditions were chosen allowing alive cells to escape from the device. In particular, the following values were chosen for the boundary conditions (5), according to the experimental results and the symmetry of the experiment, we select $K_1(x^*=0,L,t) =1$, $g_1(x^*=0,L,t)= h_1(x^*=0,L,t)=0$ and $J_1(x^*=0,L,t) =1.0 \times 10^6 \; \mathrm {s/cm}$. Only the parameter $J_1$, explaining cell leakage at boundaries, was fitted to capture the corresponding results. For dead cells, homogeneous Neumann conditions were established, assuming no migration of dead cells through the boundaries, that is, $K_2(x^*=0,L,t) =0$, $h_2(x^*=0,L,t)=0$ and $J_2(x^*=0,L,t) =1.0 \; \mathrm {s/cm}$. With respect to oxygen concentration, Dirichlet boundary conditions were chosen, so oxygen pressure in the channels was assumed to remain constant throughout the whole experiment, since medium flow provision through the channels was sufficiently frequent to keep that pressure without important variations despite oxygen diffusion and cell uptake. With that, we write $K_0(x^*=0,L,t) =1$, $g_0(x^*=0,L,t)= 7.0 \; \mathrm {mmHg}$ and $J_0(x^*=0,L,t) =0$. Finally, as initial conditions, we assume that the initial monitoring of the process starts after getting a uniform oxygen pressure in the whole chamber, equivalent to the one present in the lateral channels, that is $O_2(t=0) = 7 \, \mathrm {mmHg}$.

A heuristic approach was followed to fit the simulated curves with the experimental results, in order to determine the model parameters. This approach tried to get the best fit of the necrotic core (central region) due to its biological relevance, giving less importance to the fitting around the boundaries since the cell distribution here is not representative of the in vivo situation. To quantify the quality of this fitting procedure, we defined two objective cost functions:

22 $$\begin{aligned} T&= \frac{1}{C_{\mathrm {sat}}}\sqrt{\frac{1}{2L}\sum _{j=1}^2\sum _{i=2}^5\int _{0}^L\left( u_j(x,t_i)-u_j^e(x,t_i)\right) ^2 dx} \nonumber \\ D&= \frac{1}{C_{\mathrm {sat}}}\max _{i=2,\ldots ,5}\max _{x\in [0;L]}|u_j(x,t_i)-u^e_j(x,t_i)| \end{aligned}$$

where $u_j^e$ is the experimental measurement of the *j* phenotype ($j=1$, alive cells, $j=2$, dead cells), $u_j$ the simulated one and *L* the chip length.

**Figure 5:**
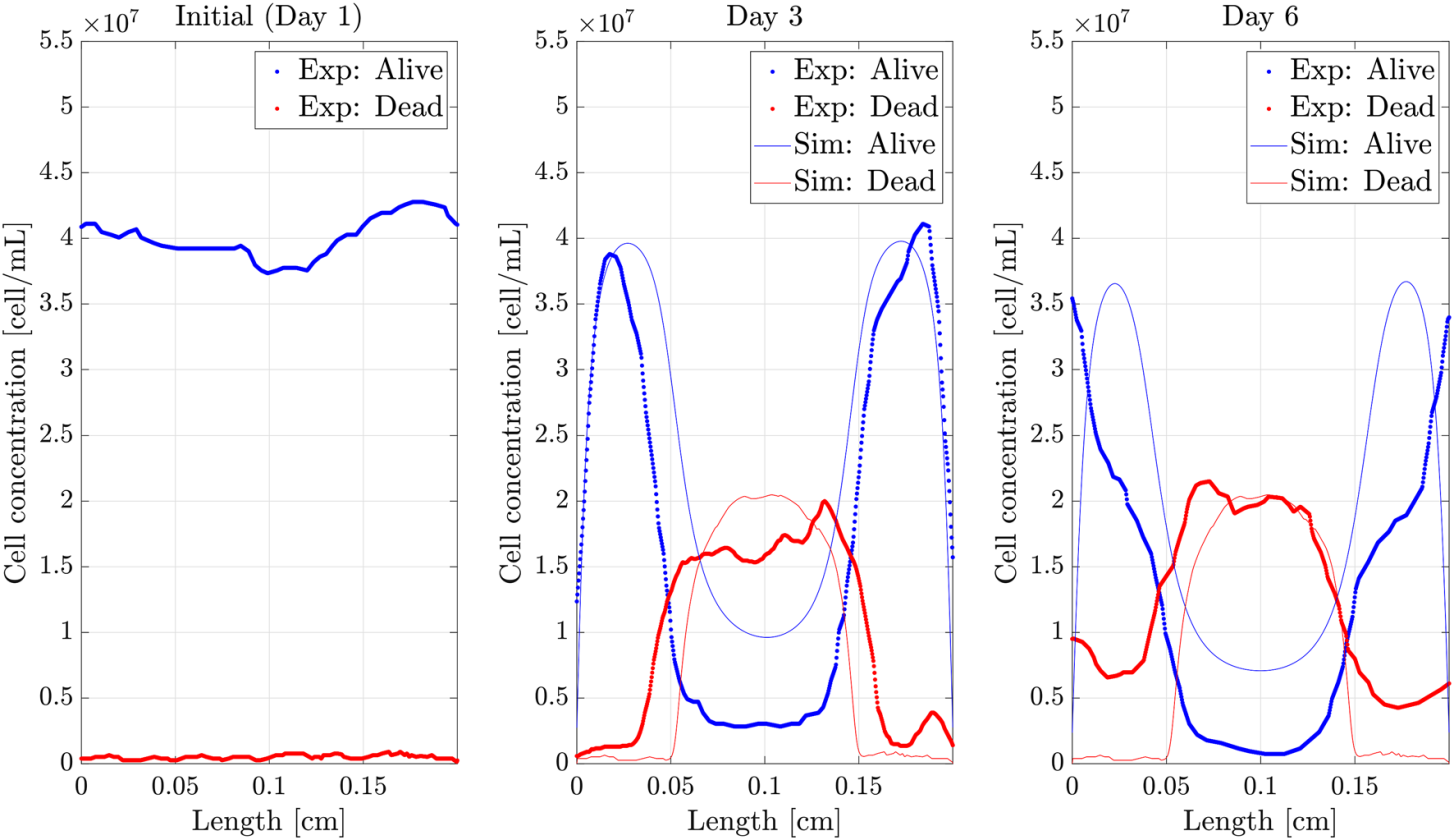
Cell concentration profiles for the defined value set. Dead and alive profiles (*y*-axis) along the length of the chip (*x*-axis) with the parameters shown in Table 2. *Sim:* Simulated profiles, *Exp:* Experimental profiles. At Day 1, both profiles coincide.

After the fitting process for the different results got in the necrotic core experiment, we arrived to the value set for the parameters shown in Table 3, yieliding the results shown in Fig. 5. The fitting process provided values of T = 0.17 and D = 0.73, which reinforce the good agreement between the experimental and simulation results.

As it may be observed, the computed results are qualitatively equal and quantitatively very similar to the experimental ones, although there are some significant discrepancies in the alive cell profile, mainly at the centre of the chamber and in the dead cell profile at the boundaries. These differences are unavoidable due to the effects and interactions missed in the model, such as the heterogeneous distribution of hydrogel, cells and oxygen in the initial state and boundary conditions, and to the highly non-linear character of the equations. As it can be seen in Table 2, the range of variation of the parameters in the bibliography is extremely wide in some cases, being therefore tricky to tune the value of the parameters to obtain better numerical results. All this makes essential to perform a sensitivity analysis to understand the quantitative impact of each parameter on the representative results and to assess the mathematical model robustness with respect to the parameter fitting.

#### Sensitivity analysis

This sensitivity analysis is performed subsequently, with the aim of defining a robust methodology capable of providing a sufficiently accurate reproduction of different in vitro experiments, while avoiding the common overfitting in biological problems.

**Figure 6:**
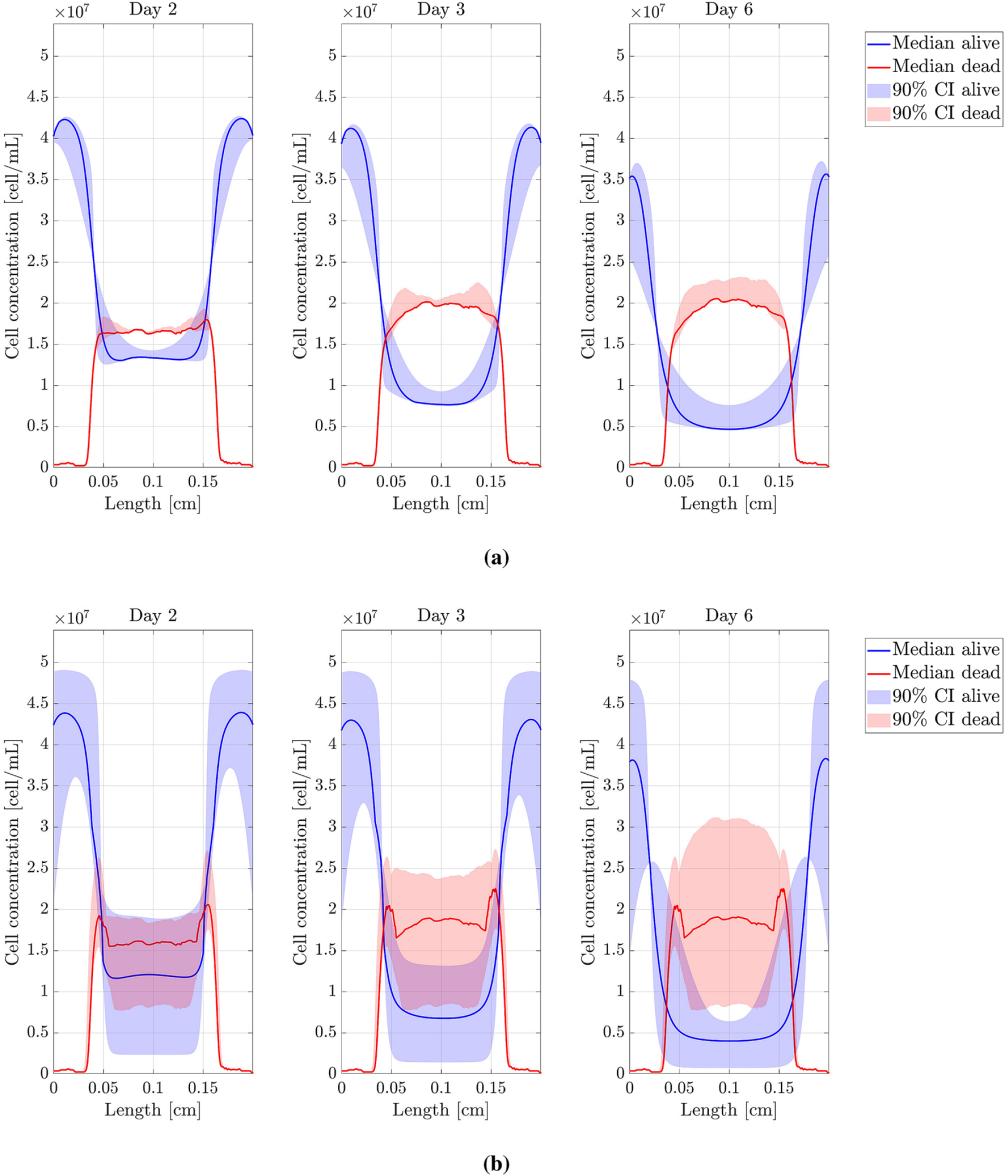
Sensitivity analysis (I). Model parameters related to cell migration. (**a**) Cell migration coefficient ($D_n$) sensitivity analysis. (**b**) Chemotaxis coefficient ($\chi$) sensitivity analysis. Figures show the cell concentration median and 90% confident interval (*y*-axis) along the length of the chip (*x*-axis).

**Figure 7:**
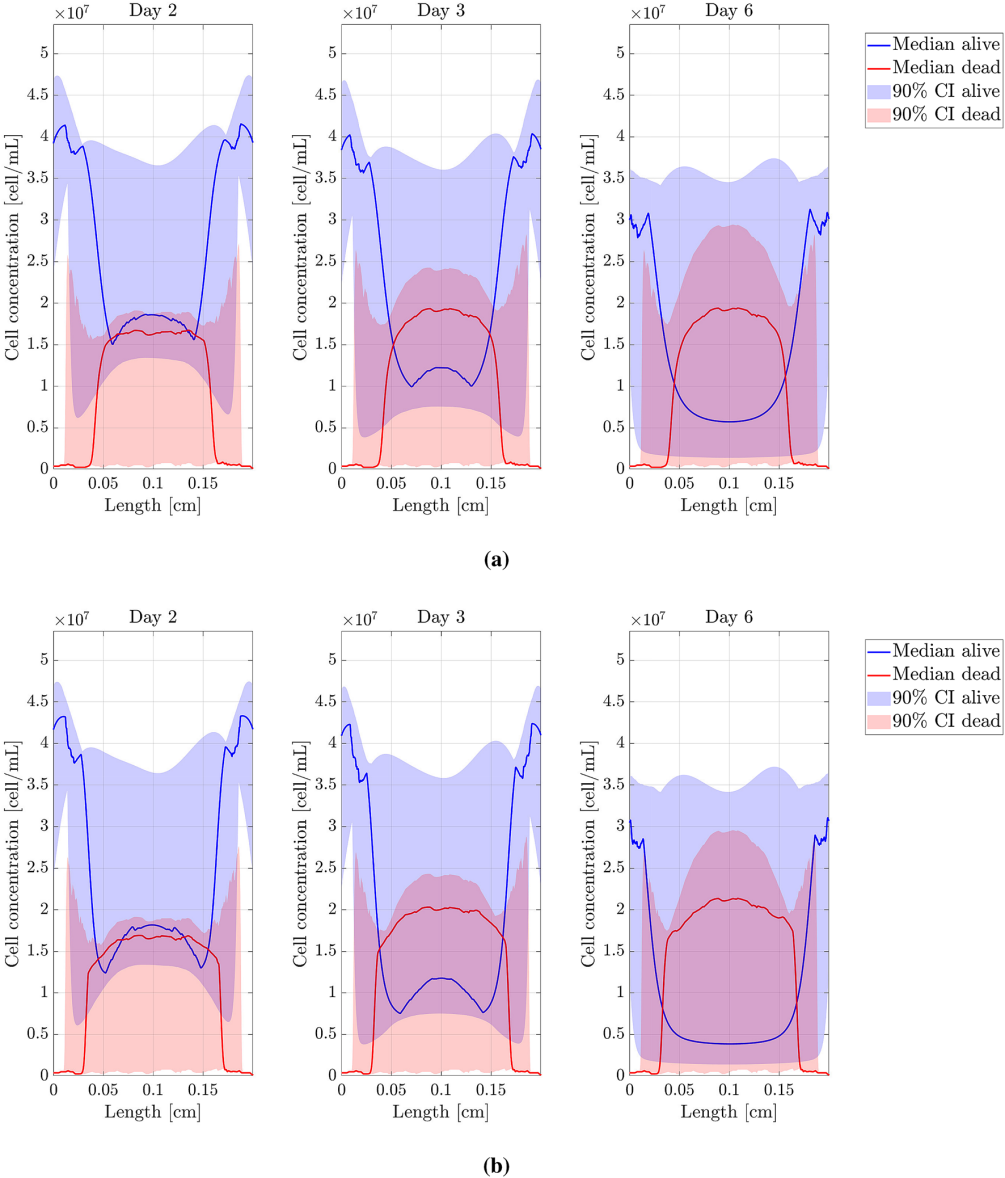
Sensitivity analysis (II). Model parameters related to oxygen evolution. (**a**) Oxygen diffusion coefficient ($D_{O_2}$) sensitivity analysis. (**b**) Oxygen uptake coefficient ($\alpha$) sensitivity analysis. Figures show the cell concentration median and 90% confident interval (*y*-axis) along the length of the chip (*x*-axis).

**Figure 8:**
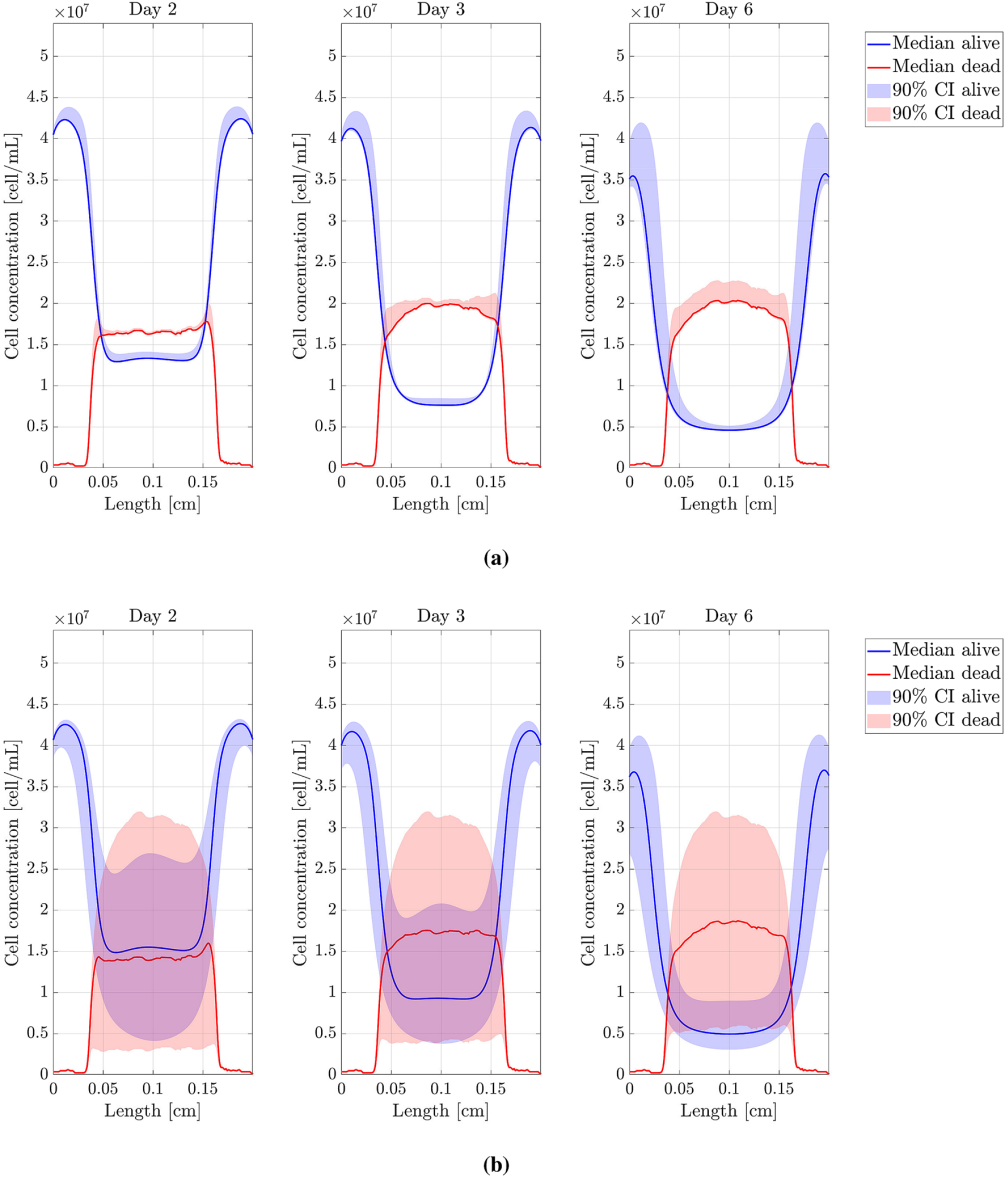
Sensitivity analysis (III). Model parameters related to cell growth and death. (**a**) Cell growth characteristic time ($\tau _g$) sensitivity analysis. (**b**) Cell death characteristic time ($\tau _d$) sensitivity analysis. Figures show the cell concentration median and 90% confident interval (*y*-axis) along the length of the chip (*x*-axis).

$D_n$, $D_{O_2}$, $\alpha$, $\chi$, $\tau _g$ and $\tau _d$ were the parameters studied. The threshold parameters were not considered in this analysis since they mainly affect the location and spread of the necrotic core. Each parameter, say $\theta$, was individually perturbed as follows: if *U* is a uniform ${\mathcal {U}}([-1;1])$ random variable, we generated 100 samples of $\Theta = \theta \times 10^{U}$, that is, $\theta$ was perturbed but maintaining its magnitude order. The non-perturbed value of $\theta$ corresponds to the value obtained after the fitting procedure. Percentiles 5, 50 (median), and 95 were extracted from the resulting concentration profiles. All results are shown in Figs. 6, 7 and 8. According to the sensitivity analysis performed, a final range of variation for $D_n$, $D_{O_2}$, $\alpha$ and $\chi$ was chosen (see Table 3), since they are the parameters with a higher impact on the computed results while presenting important variability in the bibliography. The interval bounds were selected to guarantee a value of $T<0.15$ for the best simulation in each experiment. $\tau _g$ and $\tau _d$ do not have a variation range as their impact on the results is low.

**Table 3:** Final parameter ranges. A star means that one of the bounds is out of the range found in bibliography.

Symbol	Fitted value	Simulation range	Mean value	Units
$D_n$	$6.6 \times 10^{-10}$	$5.0 \times 10^{-10}$ – $7.0 \times 10^{-10}$	$6.0 \times 10^{-10}$	$\mathrm {cm^2/s}$
$C_{\mathrm {sat}}$	$5.0 \times 10^7$	$5.0 \times 10^7$(*)	$5.0 \times 10^7$(*)	$\mathrm {cell/mL}$
$\chi$	$7.5 \times 10^{-9}$	$7.5 \times 10^{-9}$ – $15.0 \times 10^{-9}$	$11.3 \times 10^{-9}$	$\mathrm {cm^2/mmHg \, s}$
$\tau _g$	$7.2 \times 10^5$	$7.2 \times 10^5$	$7.2 \times 10^5$	$\mathrm {s}$
$\tau _d$	$1.7 \times 10^5$	$1.7 \times 10^5$	$1.7 \times 10^5$	${\mathrm {s}}$
$D_{O_2}$	$1.0 \times 10^{-5}$	$1.0 \times 10^{-5}$ – $2.0\times 10^{-5}$	$1.5\times 10^{-5}$	$\mathrm {cm^2/s}$
$\alpha$	$1.0 \times 10^{-9}$	$1.0 \times 10^{-9}$ – $3.0 \times 10^{-9}$(*)	$2.0 \times 10^{-9}$	$\mathrm {mmHg \, mL/cell \, s}$
$O_2^T$	2.5	2.5	2.5	$\mathrm {mmHg}$
$O_2^H$	7	7	7	$\mathrm {mmHg}$
$O_2^A$	1.6	1.6	1.6	$\mathrm {mmHg}$
$\Delta O_2^A$	0.1	0.1	0.1	$\mathrm {mmHg}$

For the simulations presented from here to the end of the paper, each parameter was considered as a random variable $\varvec{\Theta }$ with $\Theta _i \sim {\mathcal {U}}[\theta ^1_i;\theta ^2_i]$, with $\theta ^1_i$ and $\theta ^2_i$ in agreement with the previous discussion.

To estimate the correlation coefficients, a set of 100 simulations were performed varying the parameters within their order of magnitude (as done in the sensitivity analysis), and the sets that provide solutions with $T<0.2$ were kept. Then, the Kendall correlation coefficient was computed, obtaining a value of $\tau =0.9$ for $D_{O_2}$ and $\alpha$ and a value of $\tau =0.2$ for $D_n$ and $\chi$. The rest of the parameters are supposed to be mutually independent, and therefore uncorrelated.

A more in-depth analysis of this multiparametric correlation and its effects on the results is possible, but is out of the scope of this work. Here, the aim is to illustrate that, from the sensitivity analysis, it is possible to get a higher insight into the mathematical model that could be taken into account when performing Montecarlo simulations.

### In silico replication of the in vitro experiments

We analyse the performance of the mathematical model presented in Methods section when using one single set of parameters (Table 3), applied to the three experiments described in Methods section: formation of a necrotic core in high concentrated cultures, pseudopalisade formation due to oxygen gradient and double pseudopalisade formation in a symmetric configuration. As there is an inherent uncertainty in the parameters identification, a run of 100 Montecarlo simulations was performed for varying values of the model parameters according to the ranges defined in Table 3 and the parameter correlations discussed in the previous section.

The cells boundary conditions remained the same in all the experiments except for the value of *J*, which depends on the cell leakage observed for each microfluidic device. In experiment 1, $J =1.0 \times 10^6 \; \mathrm {s/cm}$; in experiment 2, $J = 1.0 \times 10^9 \; \mathrm {cm/s}$; and in experiment 3, $J = 1.2 \times 10^7 \; \mathrm {cm/s}$. Regarding the oxygen boundary conditions, they were adapted to each experimental configuration: in the formation of the double pseudopalisade they were identical to those already explained for the necrotic core formation; whereas in the pseudopalisade formation, impermeability condition (no flux) was imposed at the sealed channel, while, again, the Dirichlet condition was imposed at the right channel. The value of the oxygen pressure, both at the right side and at the initial time for the whole chamber, was fixed to $O_2^* = 2 \, \mathrm {mmHg}$ instead of $O_2^* = 7 \, \mathrm {mmHg}$ as in the other experiments. This is justified by the fact that in this experiment the medium was not renewed as in the previous cases, so the oxygenation was assumed to be lower.

**Figure 9:**
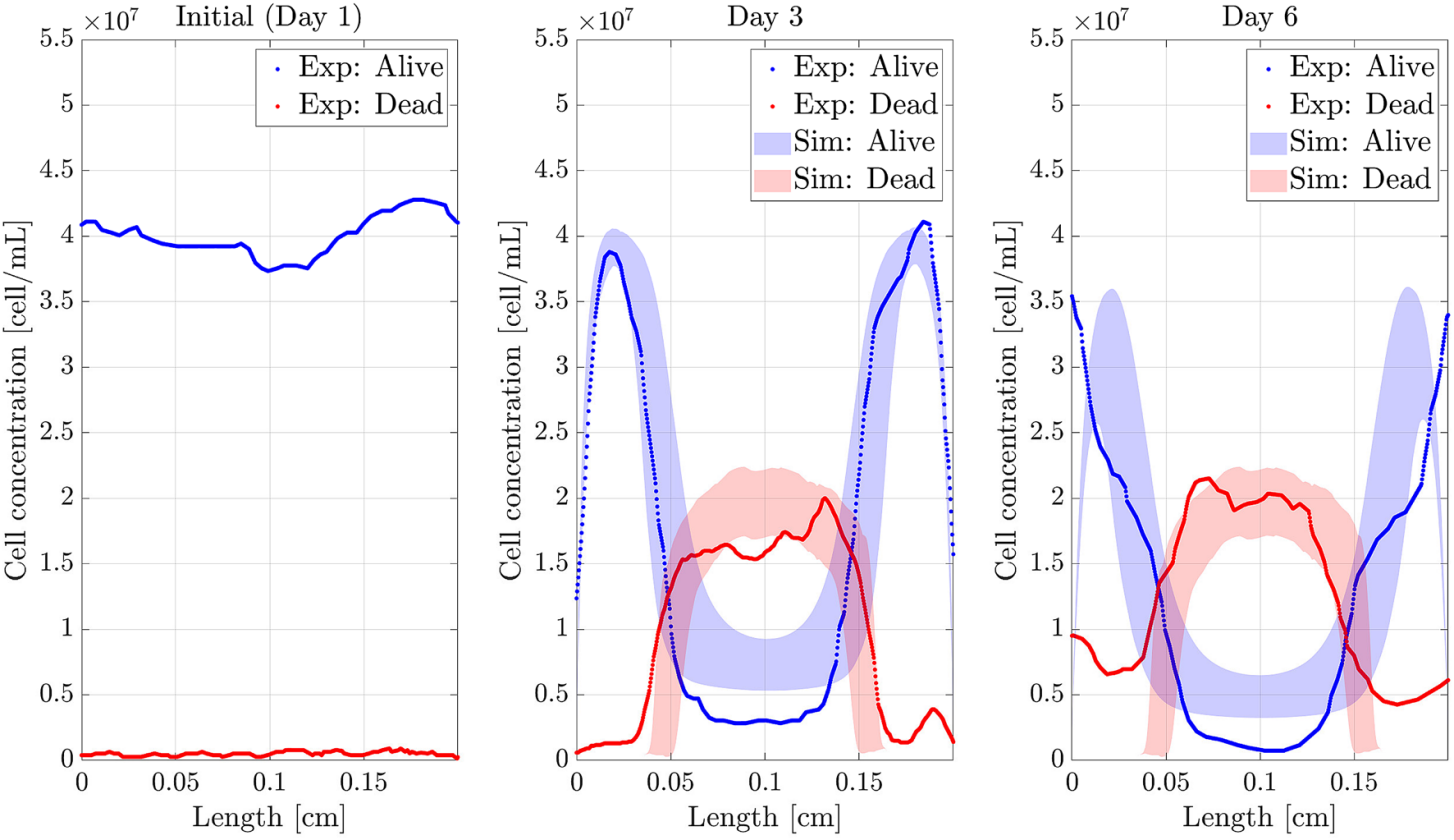
Necrotic core simulation. Confidence band of the simulated profiles and experimental results (*y*-axis) along the length of the chip (*x*-axis) for the necrotic core formation experiment. *Sim:* Simulated profiles. *Exp:* Experimental profiles.

**Figure 10:**
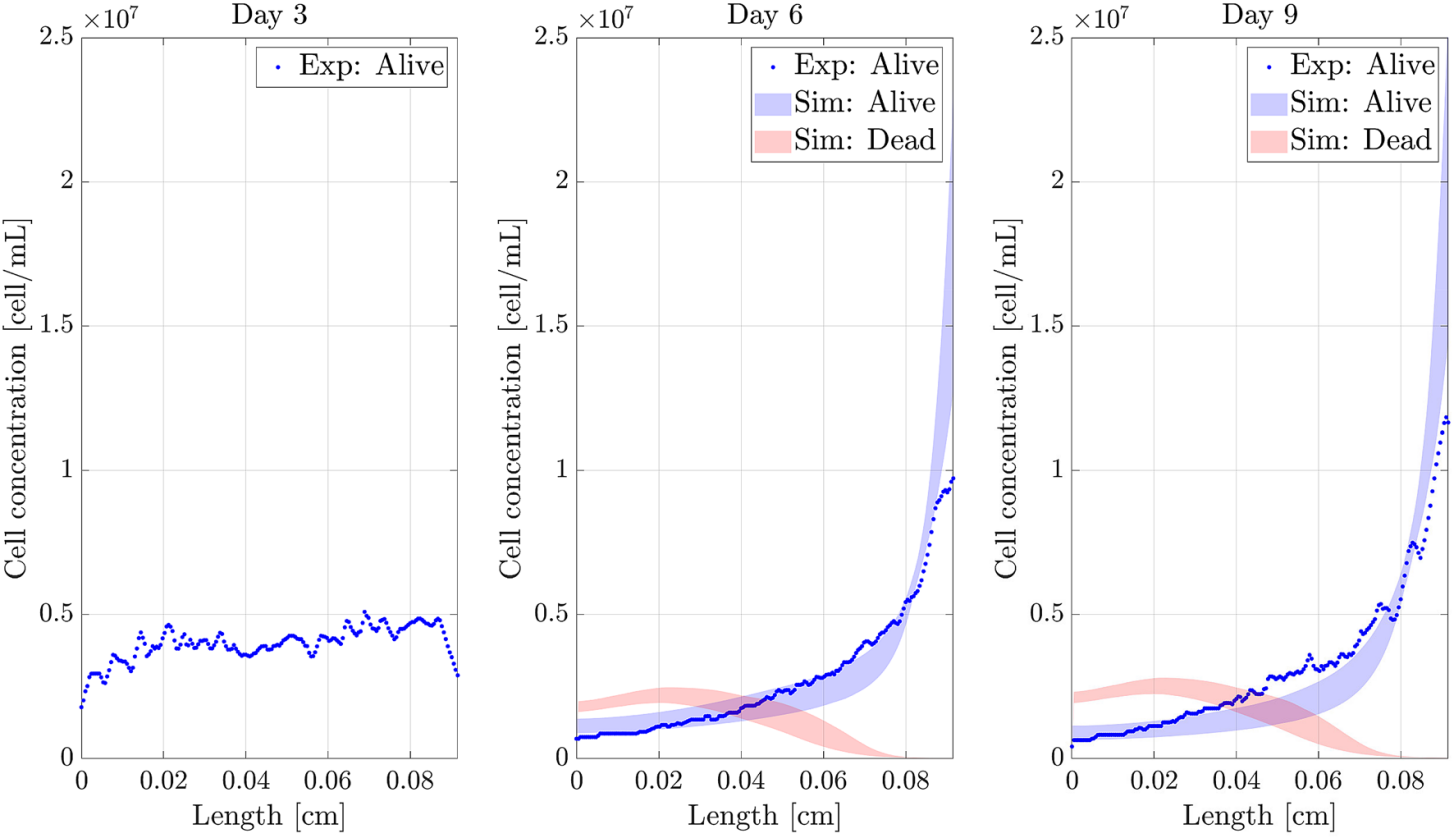
Pseudopalisade simulation. Confidence band of the simulated profiles and experimental results (*y*-axis) along the length of the chip (*x*-axis) for the pseudopalisade formation experiment. *Sim:* Simulated profiles. *Exp:* Experimental profiles.

**Figure 11:**
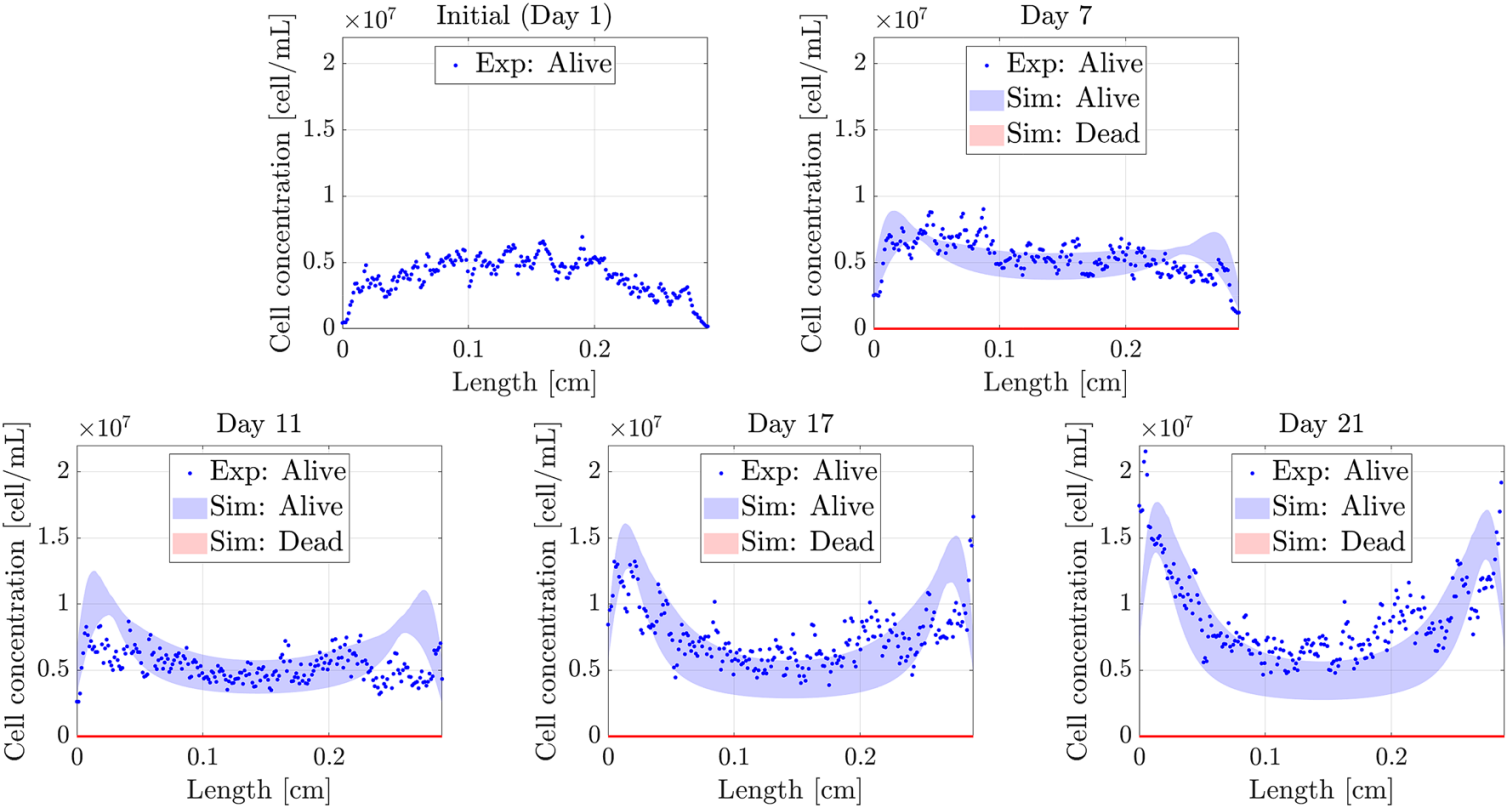
Double pseudopalisade simulation. Confidence band of the simulated profiles and experimental results (*y*-axis) along the length of the chip (*x*-axis). *Sim:* Simulated profiles. *Exp:* Experimental profiles.

The obtained results for each experiment together with the 90% confident band (between 5th and 95th percentile) of the simulations, are shown in Figs. 9, 10 and 11.

The figures show good agreement between the experimental and simulated results, which will be further explained in Discussion section. Moreover, considering the parameter variability improves the estimation of the dead cell profile (Fig. 9) .

## Discussion

Glioblastoma is one of the deadliest tumour types, as it is very heterogeneous and resistant to therapy^97–99^. Most research studies have been performed in 2D models, on Petri dishes, but these are not able to represent the real situation. Many 3D culture models are now being developed, such as spheroids, organoids, different scaffold-based models, etc., which better mimic cell-cell and cell-extracellular matrix interactions^100,101^. With the development of microfluidics for biological applications, apart from including different components of the tumour microenvironment, we are also able to control the physico-chemical conditions, so microfluidic models are considered the most biomimetical in vitro models nowadays^102,103^. Special devices have been developed for GBM research to study the behaviour of GBM cells and therapy response within a biomimetic and controlled microenvironment. With them, realistic behavioural patterns have been obtained, similar to the ones observed in patients^46,104–106^. In our case, we were able to reproduce autoinduced necrotic core and pseudopalisade formation^45,46^, some of the most important characteristics of GBM, in agreement with the GBM formation model of Brat et al.^107^, which identifies blood vessel occlusion and subsequent hypoxia-induced migration towards the functional blood vessel, following oxygen and nutrients gradient, as one of the main drivers of GBM invasion. From our models of pseudopalisades and necrotic core formation in GBM in vitro models, we set the following phenomena in the model mathematical equations:

- GBM cells migrate in an $O_2$ pressure gradient following a chemotactic cue from lower to higher pressure values, and with a migration speed that depends on the specific local $O_2$ pressure value.
- Very high cell concentrations prevent the arrival of sufficient oxygen to regions far from oxygen provisions sources, due to the oxygen uptake in the transition zones, thus resulting in an auto-induced necrotic core in those far regions. Lee et al.^108^ explained the importance of shortage of oxygen and nutrients in necrotic core formation. Also, a similar process was observed in spheroid cultures^109,110^, where the gradient depends on the spheroid diameter, observing the appearance of necrotic core in spheroids bigger than 500 $\mu \mathrm {m}$.
- Regarding the parameters of the model, we have shown that:
  - The variation of cell motility coefficient, $D_n$, has a limited impact on the results. The chemotaxis coefficient, $\chi$, has a more significant impact. Besides, the effect of both variations in the resulting cell profiles is similar. In other words, from a statistical point of view of parameter fitting, they are highly correlated.
  - The oxygen diffusion, $D_{O_2}$, and oxygen consumption, $\alpha$, coefficients have a greater impact on the results and cause both qualitative and quantitative changes. Once again, their effect on the results is similar, showing statistical correlation from a parameter fitting point of view.
  - The effect of growth and death times, $\tau _g$ and $\tau _d$ respectively, have an obvious impact over the duration and speed of the phenomena involved. However, as the chamber is essentially under hypoxic conditions, the death time plays a major role on the density of the necrotic core (but not in its size).
- Cell adaptation may also have an important role in problems like the one described here but has not been considered so far in our work. This will be part of future developments, although it will require new and specific sets of experiments to capture the corresponding mathematical features and parameters.

Since the model parameters fitting was established under a heuristic basis according to our research experience, the values selected should be interpreted qualitatively as the ones which better describe the most relevant phenomena that take place in glioblastoma evolution in vitro, such as necrotic core formation. It is important to remark that a preliminary fitting approach based on a formal mathematical optimization did not provide the best fit in the evolution of the necrotic core (data not shown), demonstrating the difficulty of the problem.

Despite the parameter uncertainty, it has been possible to reproduce three different experiments with one single set of parameters. Therefore, it seems possible to extract fundamental biological conclusions such as:

- The initial cell density has a crucial influence on the necrotic core formation. The simulation results (and the experimental curves) related to the necrotic core and the double pseudopalisade experiments are essentially identical except for the fact that the former was obtained with an initial concentration of $C_0 = 40\times 10^6 \; \mathrm {cells/mL}$ and the latter with $C_0 = 4\times 10^6 \; \mathrm {cells/mL}$. The corresponding results are however qualitatively very different: the necrotic core only appears when having very high cell concentrations. This conclusion may have important biological and therapeutic consequences.
- Cell migration depends strongly on the oxygen level and gradient. Suitable oxygenation conditions do not induce cell migration even for high oxygen gradients. Conversely, under a certain oxygenation level, the oxygen gradient is the main driving agent of cell migration. It has been shown^111,112^ that hypoxic conditions lead to stabilization of hypoxia inducing factor (HIF) which regulates many important pathways important for tumour progression, such as invasion and angiogenesis.

The presented mathematical model is, therefore, able to capture some of the main features of some essential phenomena occurring during GBM invasion. Moreover, except for the saturation parameter $C_{\mathrm {sat}}$ (that is obviously very dependent on the experimental conditions) and for some values of the oxygen consumption $\alpha$, the parameter variability is in agreement with that found in the scientific literature. Also, most of the general features observed are similar to the ones obtained in previous experimental^45,46^ and computational^41,45,48,64,71^ works.

However, the model also presents some limitations, some of the most important are the following:

- Small discrepancies between the experimental and computational results were found. One possible explanation is that the accumulation of cells at the boundaries may obstruct the oxygen diffusion, provoking a non-homogeneous $O_2$ diffusion coefficient. This results in an over-estimation of the oxygen level in the central area of the chamber, which could explain the over-estimation of alive cells, faster cell migration to the boundaries and over-estimation of dead cells.
- There is, in general but mainly in the results associated to the necrotic core experiment, a certain lag between the computational and the experimental responses of cells to oxygen variations. It seems that changes in the oxygen concentration are felt earlier in the simulations. This may be associated with a certain cell memory: the cell may need to accumulate some *damage* before undergoing significant changes in its behaviour. Our GBM model is not able to capture this kind of phenomena. Nonetheless, we have presented a framework where this limitation can be overcome if more phenotypes are considered as in other works^45,48,64^. This strategy requires, however, a sound classification of the cell phenotypes, based on biological evidence, in a sufficient number of classes, which is difficult and would considerably increase the number of parameters.

## Conclusions

From the results and discussion presented above, we enumerate the main findings and conclusions of the paper:

1. Mathematical modelling and the corresponding computer simulation of complex cell processes, incorporating cell interactions, chemical and physical cues, require an extensive literature review and the analysis of the fundamental properties of the mathematical equations in simplified conditions, and an in-depth analysis of the model parameters, in order to understand the individual and combined effect of each combination of parameters, both qualitative and quantitatively, in the resulting variables.
2. One single type of experiment is not enough to guarantee a proper quantification and understanding of the effect of each model parameter. Some families of experiments have to be used to fit the parameters, while other families are required to validate and discard spurious parametric solutions. This strategy is fundamental to avoid overfitting and to prevent model-induced effects, result of the fitting procedure.
3. There is a huge variation in the range of many of the parameters found in the literature, sometimes with intervals covering several orders of magnitude, which makes it very difficult to get a reasonable accuracy when modelling experimental tests with the only use of values from bibliography. This can be a result of the high heterogeneity of GBM and of the high adaptive capacity of these cells^113,114^.
4. With a proper parameter identification and analysis, if all the main mechanisms involved are properly considered, it is possible to get an accurate reproduction of experimental tests, provided the experiment is well controlled, the associated variables are accurately measured and the results are correctly interpreted.
5. A proper parameter sensitivity analysis is essential to discover hidden effects that cannot be explained by the model (e.g. presence of dead cells close to the lateral channels), disregard wrong value intervals (e. g. the range of the parameters was strongly reduced from Table 2 to Table 3) and identify the actual conditions in which the experiment is performed.
6. Adopting the presented model as a starting point, there is still room for future development. For instance, the measurement of the oxygen profile would allow us to improve the oxygen diffusion model, taking into account, for example, the oxygen flow obstruction that may be induced by high cell densities.

To summarise, the presented framework is general and allows the analysis of many coupled and highly non-linear physical mechanisms. The effort made in the parametric analysis allows to draw conclusions both qualitative (e.g. pseudopalisade and necrotic core formation) and quantitative (e.g. time scale for necrosis or speed of migration structures). This task is fundamental when working with complex multiparametric models. Nonetheless, this analysis is always conditioned by the choice of the mathematical approach, so the intrinsic model uncertainty is, to some extent, unavoidable. Working with models with so many parameters requires always enough experimental data in sufficiently varied conditions. There, we have been able to work with three different families of experiments resulting in cell profiles along space and time. This extensive amount of information gives value to our work, which could lay the foundations for future works in the topic.

Finally, we can conclude that, even with all this, we are still far from getting sufficient knowledge of all the mechanisms involved in complex biological processes, as well as the interactions and quantification of the corresponding phenomenological parameters. Only in very specific and well-controlled conditions, and after an extensive analysis of the tests, model and associated parameters, it is possible to expect for accurate results if the initial conditions are well-measured and the main mechanisms and interactions are mathematically represented. Despite these drawbacks, mathematical models are today invaluable tools to better understand underlying mechanisms and interactions, to establish trends, to test new hypotheses and to check “what if” situations that are many times impossible to test experimentally due to the impossibility to isolate single effects, measure particular variables or simply for ethical reasons.

## Data Availability

The codes are written in Matlab software and are available under request to the authors.
